# Triphasic Response of Pituitary Stalk Injury Secondary to Traumatic Brain Injury

**DOI:** 10.7759/cureus.87060

**Published:** 2025-06-30

**Authors:** Andrej M Sodoma, Nicholas S Bulba, Mark Baginski, Neelofar Khan

**Affiliations:** 1 Internal Medicine, Northwell Health, New Hyde Park, USA; 2 Gastroenterology, New York Institute of Techonology College of Osteopathic Medicine, Old Westbury, USA; 3 Nephrology, Northwell Health, New Hyde Park, USA

**Keywords:** acute hypernatremia, acute hyponatremia, moderate/severe tbi, pituitary hormones, syndrome of inappropriate secretion of antidiuretic hormone (siadh)

## Abstract

Traumatic brain injuries (TBIs) cause damage to the brain. Various brain structures can be vulnerable during acceleration-deceleration accidents, such as, in some cases, the pituitary organ. We present the case of a 21-year-old male with no past medical history who came to the ED of a tertiary care center after a motor vehicle accident (MVA). The patient was an unrestrained passenger ejected through the windshield of a car; he developed fluctuating sodium levels. This case demonstrates a rare occurrence of the triphasic pituitary response secondary to stalk injury, consisting of arginine vasopressin deficiency (AVP-D), syndrome of inappropriate antidiuretic hormone secretion (SIADH), and again AVP-D in a patient post traumatic brain injury (TBI). Clinical symptoms vary at each stage of the response, with management tailored accordingly. The patient in this case was admitted and diagnosed with AVP-D, initially being asymptomatic, followed shortly by hypernatremia and polyuria. In the first and last stages of AVP-D, the patient was treated with fluids and desmopressin. During the SIADH stage, the patient experienced net fluid negative and hyponatremia that was treated with fluid restriction, 3% normal saline, and conversion to salt tablets. Maintaining normal sodium levels in TBI patients is essential for preventing damage from rapid changes in osmolarity. In this case, we highlight the importance of close monitoring in the titration of sodium levels and present a successful treatment of the triphasic pituitary response secondary to stalk injury in a TBI patient.

## Introduction

The pituitary gland is a vital organ in the sella turcica, comprising two separate lobes [1]. The anterior lobe, adenohypophysis, makes up 80% of the pituitary and is a remnant of Rathke’s pouch, secreting hormones that enable actions through the hypophyseal portal system [1,2]. The hypothalamus synthesizes hormones that are connected to the posterior pituitary lobe via a connection of neurons [1]. The posterior pituitary lobe stores and secretes antidiuretic hormone (ADH) and oxytocin as needed. These intricate structures connected by vasculature and delicate neurons are at risk of damage during TBI, indicating the need for consistent monitoring. The triphasic response of the pituitary organ follows three phases of ADH dysfunction, initially with arginine vasopressin deficiency (AVP-D), followed by SIADH, and ending with AVP-D. 

The triphasic response is rare and occurs after pituitary manipulation, seen in surgery, commonly in children after craniopharyngioma resection, and pituitary injury by trauma. One report studying the triphasic response in pediatric craniopharyngioma resections found the rate to be 26% in those undergoing infundibular transaction [3]. Other studies also found the response in 14 of 117 pediatric patients and six of 21 pediatric patients [3]*.* One study focusing on TBI patients supports the theory of underdiagnosis of post-traumatic AVP-D and SIADH. Agha et al. report a study of 50 TBI patients with a median age of 35 admitted to the neurosurgical intensive care unit, with follow-up data at 6 and 12 months [4]. Thirteen (26%) of patients had AVP-D in the immediate phase (during the in-hospital stay) of TBI, with nine patients recovering by 12 months, 10 patients recovering at 12 months, and three patients with permanent AVP-D. On the contrary, seven (14%) patients had SIADH in the immediate phase of TBI, with all recovering by 6 months [4].

Mechanistically, the process occurs due to a transient shock of the pituitary, followed by a robust preformed ADH release, and ends due to a depletion of ADH secondary to neuron damage [5]. We present a unique case of a pituitary triphasic response in a 21-year-old male sustaining a traumatic brain injury (TBI) after a motor vehicle accident (MVA) with ejection through the windshield and loss of consciousness. The patient was successfully treated and managed during each stage of the response, following set guidelines and demonstrating effective monitoring and titration of serum sodium and urine output.

## Case presentation

**Figure 1:**
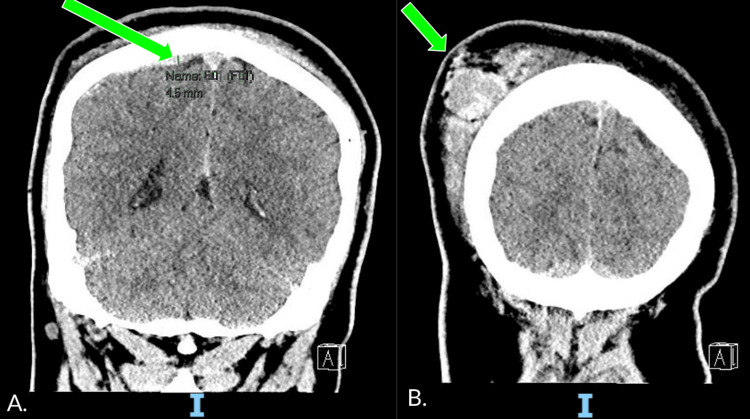
CT head without IV contrast performed on admission A. CT head showed a thin, acute high right parietal convexity subdural hematoma measuring up to 4-5 mm in maximum thickness, pointed out by the green arrow. There is no significant mass effect. B. Large right parieto-occipital scalp hematoma, pointed out by the green arrow.

EMS brought a 21-year-old male with no pertinent past medical history to the ED of a tertiary care center from the scene of a car accident. The patient was an unrestrained passenger ejected through the windshield of the car. He arrived with multiple signs of trauma, comprising lacerations to the face, arms, and legs, but no signs of neurologic deficits on further exam. The patient was alert and oriented to person and place, and was able to answer simple questions. The patient underwent a CT scan of his head and face (Figure 1), which showed a thin, acute right parietal convexity subdural hematoma measuring 4-5 mm and a large right parieto-occipital scalp hematoma. All other imaging showed no acute fractures. A set of general laboratory tests was performed upon admission (results: Tables 1-3). 

**Table 1:** Vitals on Admission

Parameter	Patient Value	Normal Range
Blood Pressure (mmHg)	132/94	<120/<80
Heart Rate (bpm)	98	60-100
Respiratory rate (rpm)	17	12-20
Temperature (F)	98.0	97-99
Oxygen Saturation (%)	98	95-100

**Table 2:** Basic Metabolic Panel on Admission

Parameter	Patient Value	Normal Range
Sodium (mEq/L)	142	135-145
Potassium (mEq/L)	4.6	3.5-4.5
Chloride (mEq/L)	105	96-106
Bicarbonate (mEq/L)	20	22-29
Anion Gap (mEq/L)	17	4-12
Blood Urea Nitrogen (mg/dL)	19.5	6-24
Creatinine (mg/dL)	0.99	0.5-1.1
Glucose (mg/dL)	111	<140 (Random)

**Table 3:** Complete Blood Count on Admission

Parameter	Patient Value	Normal Range
White Blood Cell Count (cells/uL)	12,200	4,000-11,000
Hemoglobin (g/dL)	16.3	12-15.5 (Women)
Hematocrit (%)	47.2	36-48 (Women)
Platelets (mcl)	282,000	150,000-450,000

After the patient's sodium and mentation improved, the patient was downgraded from the intensive care unit. However, the patient was polyuric, with a range of 800-1200 ml/hr overnight. He was drinking 500 ml/hr with maintenance fluids of 200 ml/hr of Ringer's lactate (LR) to keep up with his fluid requirements, as after 12 hours, the patient had a net fluid deficit of 1200 ml. A repeat set of blood tests was done, and an acute rise in his sodium level was observed (Table 4).

**Table 4:** Basic Metabolic Panel the Following Day

Parameter	Patient Value	Normal Range
Sodium (mEq/L)	150	135-145
Potassium (mEq/L)	4.6	3.5-4.5
Chloride (mEq/L)	115	96-106
Bicarbonate (mEq/L)	20	22-29
Anion Gap (mEq/L)	15	4-12
Blood Urea Nitrogen (mg/dL)	12.1	6-24
Creatinine (mg/dL)	0.97	0.5-1.1
Glucose (mg/dL)	203	<140 (Random)

The patient was completely asymptomatic. He was determined to have an acute change in sodium with euvolemic hypernatremia (Table 5).

**Table 5:** Serum and Urine Osmolarity on Second Day

Parameter	Patient Value	Normal Range
Serum (mOsm/Kg)	317	285-295
Urine (mOsm/Kg)	167	50-1200
Urine Sodium (mEq/L)	< 30	20-220

Desmopressin 2 mcg was administered IV, resulting in an improvement in serum sodium levels. He was given 0.2 mg of desmopressin orally the following day and 0.1 mg the day after. The patient continued to have polyuria and remained on IV maintenance fluids of 100 ml/hr of LR, but the amount he drank decreased. Over the course of three days, the sodium levels returned to a normal range, and the maintenance IV fluids were discontinued. The day after the last dose of desmopressin, sodium levels returned to low levels and continued to drop, reaching a nadir of 119. The patient had a net fluid status of negative 1.3 L with an average urine output of 100 ml/hr. The patient was upgraded to a higher level of care for closer monitoring of his sodium levels, and a repeat set of serum studies was ordered. The serum osmolarity was 266 mOsm/Kg, urine osmolarity of 815 mOsm/Kg, and urine sodium >250 mEq/L, which was determined to be the syndrome of antidiuretic hormone (SIADH). Also, the patient was taken to a higher level of care due to the drastic changes in sodium from 150 to 119 mEq/L over a few days, which put him at high risk for cerebral edema; neurologic checks were performed every 2 hrs, but no acute changes were observed. No repeat imaging has been performed since the admission. The patient was bolused with 100 ml of 3% sodium chloride and placed on 50 ml/hr of 2% sodium chloride for 6 hours, followed by another bolus of 3% sodium chloride with sodium acetate in a 50:50 ratio. The patient improved to a sodium level of 135 mEq/L. The patient was eventually converted to sodium tablets, 1 g every 8 hrs, and discharged after 2 days of stable sodium levels.

The patient was discharged home with a follow-up appointment with his primary care provider (PCP), with no complaints from the patient and no repeat laboratory tests performed. At the repeat appointment with his PCP, sodium was 138 mEq/L, and the patient was taken off sodium tablets. 

## Discussion

Traumatic brain injuries affect short- and long-term outcomes for patients depending on the mechanism of injury and other factors. A study analyzed 2.8 million TBI emergency department-related visits, hospitalizations, and deaths in 2013 [6]. The most common mechanisms of injury were falls, being struck by objects, and MVA [6]. Among the age groups of 15-24 and 25-34 years, the most common mechanism of TBI was MVA, correlating with the patient presented in our case [6]. In TBI, the risk of developing acute AVP-D is closely associated with the severity of damage, as assessed by the Glasgow Coma Scale (GCS), and the presence of cerebral edema on neuroimaging [7]. The patient in our case report presented with a GCS of 14 and on CT presented with a scalp hematoma and a right parietal subdural hematoma without mass effect. The risk of developing acute AVP-D in the period immediately after TBI is considered transient and ranges between 3-50% of patients, while SIADH occurred in 13-15% [7,8]. Although AVP-D and SIADH are independently reported in TBI, specific rates of the triphasic response in TBI are lacking, signifying the rarity of this case.

Traumatic brain injuries can affect the pituitary lobe and cause damage to both the anterior and posterior pituitary. One study found the prevalence of general panhypopituitarism to be 27.5% across 13 studies and 809 patients when measured 5 months after a TBI [7]. At least 45% of patients had at least one anterior pituitary hormone disorder within 3 months, and 32% after 12 months [7]. In terms of anterior pituitary dysfunction, older age, TBI severity, and fractures of the skull were predictive factors of damage [7]. The most common hormone dysfunctions found were growth hormone deficiency, secondary hypoadrenalism, and secondary hypogonadism [7].

Many theories exist in the literature regarding the mechanism of injury to the pituitary in TBI, consisting of ischemic damage, neuroendocrine insults, and immune-related factors [2]. Initial damage to the brain results in direct mechanical injury, hemorrhage, and stalk transgression [9]. The pituitary stalk is especially vulnerable in acceleration-deceleration accidents [9]. In one study of head injuries, hypothalamic-pituitary insults were apparent in 40% of autopsy cases [9]. Stalk injury accounted for 3% of those cases, with aggregated clumps of neurophysin found in the hypothalamus [9]. The aggregation of this compound results in ineffective vasopressin secretion and denervation of the posterior pituitary lobe. Direct injuries to the pituitary can lead to secondary effects, including reduced blood flow, hypotension, and alterations to the blood-brain barrier (BBB). One theory suggests that the defect in the BBB allows antigens and antibodies to expose the pituitary gland to attack [9].

In this case, rapid acceleration and deceleration during the patient's ejection from the vehicle could have caused damage to the pituitary stalk, resulting in the interruption of blood flow to the hypothalamus and pituitary, which in turn led to the first phase of the response: a decrease in the release of ADH and a rapid rise in sodium levels. The damaged pituitary then began to degenerate, resulting in the second phase, which caused a rapid release of stored ADH and a subsequent drop in sodium levels. After this, the patient recovered. At this time, the insult to his pituitary was likely reversible and is unlikely to develop chronic central diabetes insipidus (DI) unless further injuries occur. However, the potential nuances of the underlying pathophysiology and subclinical damage make predicting the possible long-term effects difficult [10]. Hence, continued monitoring should be considered. 

As documented in prior literature, it is not uncommon to find AVP-D and SIADH occurring independently after TBI. The patient presented in our case followed a rare trajectory of undergoing the triphasic response of the pituitary, beginning with AVP-D, then SIADH, and ending with AVP-D. The mechanism behind the triphasic pituitary stalk response occurs due to a transient shock of the pituitary, causing a decrease in ADH, followed by a robust release of preformed ADH. It ends due to a deficiency and synthesis of ADH secondary to neuron damage [5]. The pathophysiology behind the initial phase of AVP-D is the deficiency of arginine vasopressin (AVP), which causes polyuria and polydipsia, leading to hypernatremia. The deficiency of AVP causes rapid electrolyte dysfunction, highlighting the need for frequent plasma and urine osmolality measurements. At this stage, serum sodium and plasma osmolality are elevated, while urine osmolality and specific gravity are decreased.

The patient in our case had a peak serum sodium of 150 mmol/L during admission and 145 mmol/L on day 10. One retrospective study focusing on serum sodium fluctuations after craniopharyngioma resection in 202 pediatric patients found an average sodium peak of the triphasic response during days 2 and 14 [10]. Management of AVP-D is initiated with fluids to combat dehydration and desmopressin. Some cases have reported that fluid replacement alone is sufficient to correct mild hypernatremia (<150 mmol/L) [5]. When severe hypernatremia is present, it serves as an individual risk factor for increased mortality [5]. The patient in our case was treated with IV fluids and desmopressin, improving his serum sodium concentrations. The goal of correcting hypernatremia is to reduce the urine output as much as possible without overcorrecting the sodium [5].

The next stage of the triphasic response is SIADH - the only phase that differs from the rest, requiring a distinct management plan. This phase begins due to the uncontrollable release of preformed AVP remaining in the posterior pituitary. Due to the high AVP, the mechanism of the hormone increases the amount of water reabsorbed through aquaporin channels in the distal convoluted tubules and collecting ducts, resulting in small amounts of concentrated urine and diluted serum. Consequently, hyponatremia ensues, serum osmolality decreases, urine osmolality increases, and the specific gravity of the urine increases. Clinical features during this stage are typically nonspecific, characterized by euvolemic hyponatremia and the absence of peripheral edema. Symptoms such as nausea, vomiting, headaches, muscle weakness, and altered mental status can occur.

The patient in our case reached his lowest serum sodium nadir on day 5 at 119 mmol/L. A previous study** **regarding serum sodium fluctuations found its lowest nadir on day 6 [11]. To manage SIADH, all fluids are restricted to 500 mL less than the patient's daily basal fluid intake. If the hyponatremia is severe and below 120 mmol/L, or acutely hyponatremic with or without symptoms below 130 mmol/L, then 3% hypertonic saline can be initiated [12]. The patient in our case was treated with fluid restriction and 3% normal saline that was eventually converted to salt tablets. The goal is to monitor the serum sodium hourly until it increases by 4-6 mmol/L, ideally by not more than 10-12 mmol/L in 24 hours due to the risk of osmotic demyelination [12]. A mild correction of 4-6 mmol/L is enough to correct symptomatic hyponatremia and decrease morbidity and mortality from overcorrection [12]. There are various treatment options for this condition, including adjunctive therapy with urea, vaptans, demeclocycline, and furosemide, which can be used in cases of refractory disease [13]. However, positive patient outcomes still highly depend on administering fluids and desmopressin with consistent and frequent monitoring [13].

The final stage of the triphasic response is the return of AVP-D, a stage that sometimes remains permanent. The mechanism behind this stage involves neuron damage sustained during TBI and the depletion of preformed AVP, leading to posterior pituitary dysfunction. As discussed, careful monitoring of urine output, serum sodium, and osmolality is necessary to minimize sodium fluctuations. This stage is clinically similar to the first, presenting with a high serum sodium and plasma osmolality contrasted with a low urine osmolality and specific gravity.

Although the triphasic response of the pituitary stalk is mainly seen in craniopharyngioma resections in children, it has been documented in the literature in those with TBI. One case report of a 24-year-old female after an MVA and ejection demonstrated the triphasic response with an initial peak serum sodium of 160 mmol/L [14]. The patient was treated with hypotonic fluid administration, and no desmopressin therapy was needed [14]. Her other two phases followed with a serum sodium nadir of 118 mmol/L on day 7 and then a return to AVP-D after day 7 [14]. She was successfully treated with fluid restriction and high-dose oral salt loading during the second stage and oral desmopressin in the third stage [14]. The report successfully demonstrated a positive outcome and effective management of the triphasic pituitary response, utilizing laboratory and urine output monitoring. 

A different case report demonstrated the phenomenon in a 20-year-old male who struck his head on a curb [15]. He presented five days after injury with a serum sodium of 153 mmol/L and was treated with maintenance IV fluids and subcutaneous desmopressin [15]. After being discharged home, he returned with a serum sodium level of 120 mmol/L and was successfully treated with 3% hypertonic saline and a free water restriction of less than 1.5 L [15]. An abrupt increase of sodium to 137 mmol/L prompted desmopressin therapy, and the patient was allowed to drink to thirst, stabilizing his sodium levels and improving his urine output [15]. Both of these case reports align with our case of a 21-year-old male who presented with a TBI, demonstrating successful management and treatment of the pituitary triphasic response.

## Conclusions

Triphasic pituitary response secondary to a pituitary stalk injury is a well-established theory; however, it is rare to encounter it in clinical practice. Most occurrences occur in children after craniopharyngioma resections, with only a few known to have happened after TBI, as in our patient. This unique case, management, and successful treatment highlight the importance of monitoring a patient’s vital signs, electrolytes, and fluids after a traumatic brain injury. Not all patients are alert and oriented after a TBI and thus cannot effectively communicate their complaints. Coupled with the underdiagnosis of immediate AVP-D and SIADH occurring after TBI, more research needs to be done to study the long-term outcomes of the triphasic response post-TBI. With heightened monitoring of electrolytes and fluids, we can effectively manage and treat the triphasic response of the pituitary, resulting in lower morbidity and mortality.
